# Palmaris Brevis Syndrome: A Treatable Pseudodystonia

**DOI:** 10.5334/tohm.659

**Published:** 2021-10-29

**Authors:** Mark S. LeDoux, Jianfeng Xiao

**Affiliations:** 1 Veracity Neuroscience LLC, Memphis, Tennessee, USA; 2 University of Memphis, Memphis, Tennessee, USA; 3 University of Tennessee Health Science Center, Memphis, Tennessee, USA

**Keywords:** Palmaris brevis, Dystonia, Spasm, Botulinum toxin, Ulnar nerve

## Abstract

**Background:**

Palmaris brevis syndrome, a pseudodystonia characterized by abnormal involuntary contractions of the palmaris brevis muscle which resides in the hypothenar eminence, is believed to be due to compressive irritation of motor fibers which arise from the superficial branch of the ulnar nerve.

**Case report:**

Herein, we review the origins, differential diagnosis and pathophysiology of the palmaris brevis syndrome, and effective treatment of a patient with workplace modifications and injections of botulinum toxin type A.

**Discussion:**

Prompt diagnosis of the palmaris brevis syndrome facilitates effective treatment and resolution.

**Highlights:**

Like the task-specific hand dystonias seen in writers and musicians, palmaris brevis syndrome, a pseudodystonia, may be caused and aggravated by extreme repetitive use. Here, we report a case of palmaris brevis syndrome apparently triggered by high-volume use of a pipette and computer mouse and review relevant clinical facets from previously published cases. Treatment must include workplace modifications and may include injections of botulinum toxin.

## Introduction

The palmaris brevis is a thin quadrilateral muscle located over the hypothenar eminence and ulnar canal [1]. The palmaris brevis originates from the palmar aponeurosis and transverse carpal ligament and attaches to the deep dermis along the ulnar side of the palm and, occasionally, the pisiform bone [2]. The palmaris brevis is the only muscle innervated by motor fibers of the superficial ulnar nerve. The sensory fibers of this nerve innervate the small finger and medial half of the ring finger. The palmaris brevis is absent in approximately 3% of humans [3].

The palmaris brevis, a striated muscle, may play a role in protecting the neurovascular bundle within the ulnar canal, deepening the palm to facilitate grasping certain objects, and tensing the skin on the ulnar side of the palm to improve grip [4]. The palmaris brevis can be volitionally activated in concert with other muscles of the hypothenar eminence.

Palmaris brevis syndrome is characterized by spontaneous, involuntary contractions of the palmaris brevis muscle [5,6]. Electromyography (EMG) shows high frequency discharges of motor units, often prolonged and recurrent, with normal amplitude, duration, and morphology. In occasional patients, co-contraction of the abductor digiti minimi muscle has been described during portions of the spasms. Spasms may occur spontaneously or be triggered by forceful movements that require use of the hypothenar muscles. It has been postulated that the palmaris brevis syndrome is caused by damage to the superficial branch of the ulnar nerve, particularly those fibers innervating the palmaris brevis muscle. Some patients with palmaris brevis syndrome report numbness or paresthesias within the sensory distribution of the superficial branch of the ulnar nerve. Many patients report discomfort along the hypothenar eminence. Contractions of the palmaris brevis muscle produce a characteristic dimpling or rostral-caudal furrowing along the ulnar side of the hypothenar eminence. Palmaris brevis syndrome can be familial, bilateral, and occur in children [7,8].

## Case Report

**Figure 1:**
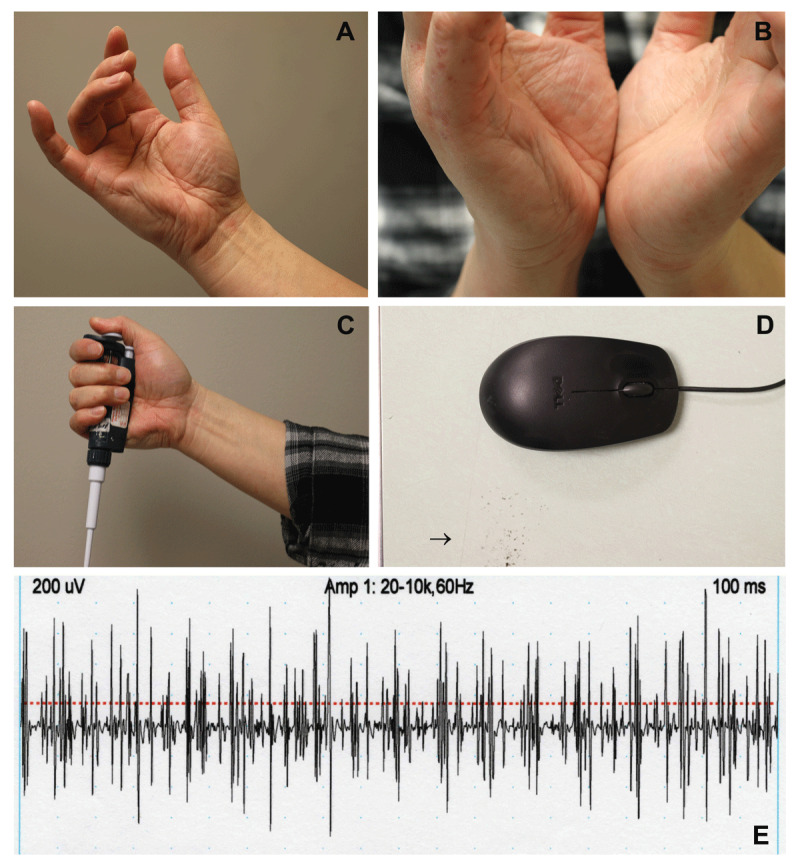
**(A)** Characteristic furrowing of the hypothenar eminence seen in our patient with palmaris brevis syndrome. **(B)** There was no visual evidence that the non-dominant hand was similarly affected. **(C)** Worsening of palmaris brevis contractions triggered by grasping a pipettor. **(D)** Patient’s workspace shows evidence of extreme wear with erosion of the countertop (arrow). He did not use a mouse pad or wrist support while handling the mouse. **(E)** EMG recording of spontaneous activity from the right palmaris brevis muscle.

A 52-year-old right-handed male was referred to our movement disorders clinic by a general neurologist for evaluation of “hand dystonia.” He reported uncomfortable, mildly painful “spasms” of the right hand for approximately 3.5 months. Although initially triggered by workplace activities with the right hand, the spasms intermittently occurred at rest within weeks of onset. At the time of neurological presentation, the spasms were present during most of the waking day and associated with dimpling or creasing of the right hypothenar eminence (***Figure 1A*** and ***B***). The patient described almost daily prolonged and repetitive use of pipettes during his work in a molecular biology laboratory. Precise pipetting of microliter and submicroliter volumes required substantial activation of the pinky finger for stabilization of the pipettor body or handle (***Figure 1C***). In addition, he routinely used a computer mouse without a mouse pad. He had worn down the countertop on the computer table where the mouse was stationed (***Figure 1D***). His almost daily use of pipettors and computer mice had been ongoing for over 15 years. Approximately four to five years prior to neurological presentation, he experienced a brief period with similar discomfort in his right hand that resolved on its own accord.

The patient reported subjective numbness in his small finger and the ulnar half of his ring finger. However, sensory examination was normal. EMG and nerve conduction studies of the upper extremities were also normal. The right median motor showed a normal distal latency of 3.1 ms, normal amplitude of 14.6 mV, and normal conduction velocity of 62.6 m/s. The right ulnar motor showed a normal distal latency of 2.7 ms, normal amplitude of 13.7 mV, and normal conduction velocity of 51.2 m/s. The right median showed a normal F wave latency of 26.4 ms, and right ulnar showed a normal F wave latency of 27.8 ms. Sensory nerve conduction examination was normal with a right median peak latency of 2.6 ms and amplitude of 60.5 μV; right ulnar peak latency of 2.3 ms and amplitude of 22.7 μV; and right radial peak latency of 2.3 ms and amplitude of 35.8 μV. Needle EMG of the right first dorsal interosseous, flexor carpi radialis, brachioradialis, flexor carpi ulnaris, biceps, deltoid, abductor digiti minimi, flexor digiti minimi brevis, and abductor pollicis brevis was within normal limits. Needle EMG of the palmaris brevis showed prolonged bursts of spontaneous motor unit activity of fluctuating amplitude (***Figure 1E***).

EMG guidance (30-gauge needle) was used for injection of 12.5 units of onabotulinumtoxinA diluted in a total volume of 0.25 cc sterile preservative free saline through three cutaneous entry sites along the proximal to distal extent of the right palmaris brevis muscle. The palmaris brevis muscle was injected at locations of maximal EMG activity which required horizontal movement of the injection needle within the subdermal space. The patient tolerated the injections without complication. He was told to purchase a larger ergonomic mouse, and mouse pad with wrist cushion. He was also instructed to wear large disposable gloves over a padded fingerless glove when pipetting and increase use of the left hand when pipetting and performing other routine laboratory duties such as opening bottles with screw tops. He reported approximately 75% improvement at 6-week follow-up. There was continued improvement over the subsequent 6 months with no need for repeat injections of botulinum toxin. His condition continued to improve over the course of the subsequent year and largely resolved within 2 years of onset.

## Discussion

The differential diagnosis of palmaris brevis syndrome includes task-specific hand dystonias, C8 or T1 radiculopathy, ulnar neuropathy, other neuromuscular disorders including myotonia, inflammatory arthritis of the wrist and Dupuytren’s contracture. Some patients have shown benefit with phenytoin [5], and precisely targeted injections of botulinum toxin [9]. The potential side effects of oral pharmacotherapy will likely outweigh benefits in most patients but may be considered in patients unwilling to receive injections. Most importantly, workplace modifications are critically necessary for long-term improvement. Every effort should be made to avoid or limit compression of the proximal, lateral hypothenar region to prevent continued irritation of motor fibers to the palmaris brevis muscle. Simply switching to the contralateral hand is not appropriate since palmaris brevis syndrome can occur bilaterally.

If necessary, injections of botulinum toxin can effectively relieve the discomfort associated with prolonged involuntary muscle contractions [9]. Injections should target the entire rostral-caudal extent of the palmaris brevis muscle and avoid underlying muscles of the hypothenar eminence. EMG or ultrasound is essential for proper localization of injections to the palmaris brevis muscle and limiting spread to subcutaneous tissues and other muscles. The starting dosage of onabotulinumtoxinA or incobotulinumtoxinA (10 – 25 units) should be dictated by spasm severity, hand size, and patient expectations.

Dystonia has been defined as a movement disorder characterized by sustained or intermittent muscle contractions causing abnormal, often repetitive, movements, postures, or both [10]. Conditions that mimic dystonia but ostensibly have different pathophysiological underpinnings are often called pseudodystonias [10,11]. “The term pseudodystonia can be used to describe abnormal postures, repetitive movements, or both, in which results of clinical, imaging, laboratory or electrophysiological investigations provide definite explanation of symptoms which is not compatible with dystonia [11]”.

Differentiation of dystonia from a pseudodystonia may be difficult, often requiring a deep understanding of regionally specific disorders associated with involuntary muscle contractions or abnormal postures. Most would agree that the likely pathophysiology of the palmaris brevis syndrome differs from the pathophysiologies of the most common adult-onset isolated dystonia (cervical dystonia) and a well-characterized childhood dystonia (DYT1 due to the classic ΔGAG mutation in *TOR1A*). Although far less than ideal, the term pseudodystonia is useful in the context of differentiating the palmaris brevis syndrome from the more common task-specific dystonias seen in adults. The absence of a sensory trick, eventual resolution with workplace modifications, and presence at complete rest helps to differentiate palmaris brevis syndrome from isolated dystonia. In the hand, dystonia, often task-specific, must be distinguished from muscle spasms secondary to electrolyte abnormalities or radiculopathies, Dupuytren’s contracture(s), trigger finger(s), isolated bone and joint abnormalities due to orthopedic and rheumatological conditions, and psychogenic disorders, including malingering. Establishing a correct diagnosis may require electromyoneurography, lidocaine block of the superficial ulnar nerve, and radiographic imaging.

## Financial Disclosures

Dr. LeDoux has been a consultant for USWorldMeds; speaker for Adamas Pharmaceuticals, Acadia Pharmaceuticals, Teva Pharmaceutical Industries, Amneal, USWorldMeds, Supernus, Kyowa Kirin, and Acorda Therapeutics; and receives publishing royalties from Elsevier (Animal Models of Movement Disorders, and Movement Disorders: Genetics and Models) and TheBookPatch (Parkinson’s Disease Poetry). Dr. LeDoux’s research has been funded by the Michael J. Fox Foundation, National Institutes of Health, Axovant Sciences, Wave Life Sciences, Teva Pharmaceutical Industries, Pharma Two B, Revance, Cerevel, Aeon, NeuroDerm, Dystonia Medical Research Foundation, and Benign Essential Tremor Research Foundation.
